# Increasing learning, confidence, and retention in the immunology classroom with LEGO

**DOI:** 10.1093/immhor/vlag043

**Published:** 2026-08-13

**Authors:** Bernice Yphantides, Heather A Bruns, Louis B Justement, Christine Loyd, Mallary C Greenlee-Wacker

**Affiliations:** Biological Sciences Department, California Polytechnic State University, San Luis Obispo, CA, United States; Department of Microbiology, University of Alabama at Birmingham Heersink School of Medicine, Birmingham, AL, United States; Department of Microbiology, University of Alabama at Birmingham Heersink School of Medicine, Birmingham, AL, United States; Department of Clinical and Diagnostic Sciences, University of Alabama at Birmingham School of Health Professions, Birmingham, AL, United States; Biological Sciences Department, California Polytechnic State University, San Luis Obispo, CA, United States

**Keywords:** antibodies, gene rearrangement, immunology education, repertoire development

## Abstract

The process of variable–diversity–joining (V(D)J) recombination gives rise to incredible diversity in the Ab repertoire. Given its complexity, V(D)J recombination is a difficult process for undergraduates to learn. We hypothesized that modeling recombination with LEGO would increase learning gains, as well as student confidence and interest in this process. To test this, students at 3 undergraduate universities watched the same video lecture about recombination, then the LEGO modeling exercise was either demonstrated in front of students or performed by students. Students who modeled or observed modeling with LEGO performed significantly better on an exam essay question than those that opted out of the modeling exercise. Our next objective was to determine whether tactile manipulation of the model was associated with increased interest and confidence. Survey results suggested that most students preferred visual learning, followed by explanations and activities. Although most students agreed that the LEGO activity gave them more confidence and increased their interest in the material, students who did the activity were more likely to strongly agree with affirming statements. For example, students who modeled were more likely to strongly agree that the LEGO activity helped them memorize the steps of V(D)J recombination and increased their level of confidence in describing key aspects of the process. Overall, these data suggest LEGO modeling of V(D)J recombination improves student comprehension, interest, and confidence.

## Introduction

The benefits of active learning over traditional passive instruction have been well documented across science, technology, engineering, and mathematics (STEM) disciplines.^1^^,^^2^ Although active learning strategies vary widely, a particularly effective approach involves the use of physical models to represent abstract and complex concepts. In the context of science education, modeling is considered a crosscutting concept that helps students identify how parts of a system interact—an approach that reinforces student comprehension, systems thinking, and scientific reasoning.^3–5^ When students physically engage with models, they can construct their own explanations, refine and reorganize prior knowledge, and identify misunderstandings and knowledge gaps. Despite their instructional power, physical models also come with limitations, including the need for dedicated class time and material costs. One way to address these challenges is to evaluate whether a guided visualization of a model can result in comparable comprehension and system-level understanding compared with direct, hands-on manipulation. These model visualizations may be especially helpful when learning topics that are invisible to the naked eye, such as protein folding or DNA recombination, and if designed intentionally, can convey complex theories with equal or greater effectiveness.^4^^,^^6^

Immunology educators recognize the need for curricular revisions and are eager to develop student-centered activities that embody a learn-by-doing philosophy.^7–9^ Immunology presents a particular challenge in undergraduate education because it introduces new systems of nomenclature, involves reactions occurring across time and space, and focuses on molecular processes that are often unfamiliar or underemphasized in prerequisite coursework.^10^^,^^11^ For example, the idea that DNA can be rearranged in somatic cells often contradicts prior learning, surprising students who have been taught that all cells in the body contain identical genetic material or that inherited genes are the primary determinants of any trait.^12^^,^^13^ This cognitive dissonance, combined with a steep learning curve for new terminology, genetic mechanisms, and spatial and temporal regulation, can overwhelm even motivated learners. The perception of difficulty may also lead to a lack of interest and confidence, feelings that may negatively influence retention in STEM.^14^ As highlighted earlier, implementing modeling offers a solution for simplifying these concepts. Consistent with the impact of modeling on learning immunology, we previously showed that using LEGO bricks to depict complement activation increased interest in the complement system and improved learning in a naïve student population.^15^

By incorporating active learning methods, educators can enhance student comprehension and retention of immunological concepts, ultimately facilitating more effective learning outcomes in the field of immunology. Given the complexity of lymphocyte receptor gene rearrangement, a process known as variable–diversity–joining (V(D)J) recombination, we developed a modeling exercise using LEGO bricks to support student understanding and to foster a greater appreciation for this immunological phenomenon. This intervention was implemented and assessed across 3 undergraduate institutions: the University of Alabama at Birmingham (UAB), Central Michigan University (CMU), and California Polytechnic State University (Cal Poly). The purpose of this study was to determine whether modeling V(D)J recombination with LEGO bricks increased learning gains, confidence, and interest among undergraduate immunology students. Additionally, we evaluated whether performing the activity or observing the model demonstration differentially influenced student exam performance, confidence, and interest in the topic.

## Materials and methods

### Participants, study design, and ethical approval

Participants in this study were enrolled in 4 different immunology courses across UAB, CMU, and Cal Poly, as noted. The courses and number of participants are summarized in Table 1. Of participants in the UAB immunology course (instructor L.B.J.), 16 students completed the LEGO modeling activity in small groups during class, whereas 17 students opted out of the modeling exercise and served as the control group. At CMU, all participants observed the LEGO modeling activity performed live under a document camera during lecture. In all cases, students were introduced to the concept of V(D)J recombination using the same prerecorded lecture video (https://youtu.be/ag5v1x9OlPU), and the activity was followed by a survey. Because it was part of the normal assessment practices, an exam question preceded the survey in the immunology survey course for juniors and seniors. Learning was assessed when participants completed an open-ended essay question on V(D)J recombination as part of their unit exam.

**Table 1:** Comparison of course and participant characteristics across institutions.

Institution	UAB	CMU	Cal Poly
**Course type**	Introduction to immunology	Survey of immunology	Survey of immunology
**Instructor**	Justement	Greenlee-Wacker	Greenlee-Wacker
**Modality**	In-person	In-person	In-person
	Lecture only	Lecture only	Lecture and lab
**Length of term**	16 wk	14 wk	10 wk
**Primary student population**	Sophomore immunology majors	Junior and senior biology majors^a^	Junior and senior in biology majors^a^
**Intervention**	Modeling or no activity	Observation	Observation or modeling
**Participants for exam, no.**	33	15	12
**Survey participants, no.**	33	15	15
**Survey timing**	5 lectures after the exam	First lecture after exam	4 lectures after exam

^a^Predominately biology majors, with a small number of biochemistry and biomedical engineering students.

At Cal Poly, students were enrolled in a single immunology course paired with 2 separate lab sections. In the first lab section, students observed the LEGO modeling activity demonstrated under a document camera (*n* = 9), and in the second lab section, students performed the modeling activity in small groups (*n* = 6). The activity was followed with an identical essay question and post-exam survey. This cohort was added after initial results demonstrated learning gains from both observing and performing the activity; therefore, a no-activity control group was not used at this site for ethical and instructional purposes. Three students in the observing group did not complete the separate identification required for linking survey responses to exam scores. As a result, only data from students whose responses could be matched to the assessment were included in the exam score analysis.

All procedures involving human participants were approved by the appropriate institutional review board (IRB). The UAB IRB approved data collection from both UAB and CMU participants under protocol no. IRB-300008092. Although the CMU study began prior to final IRB approval, CMU granted permission to use the course-based data for publication. Data collection at Cal Poly was approved under protocol no. 2024-107-CP. No identifying student data were shared between institutions, and all responses were anonymized and analyzed in aggregate.

### LEGO-based simulation of V(D)J recombination

The modeling activity was designed to help students visualize the mechanisms of V(D)J recombination by simulating key steps using LEGO bricks. Prior to the activity, all students viewed the same instructional video on lymphocyte receptor rearrangement. The LEGO-based model was then introduced either as a demonstration (observed condition) or as a hands-on activity (modeling condition), depending on institutional group assignments.

**Figure 1:**
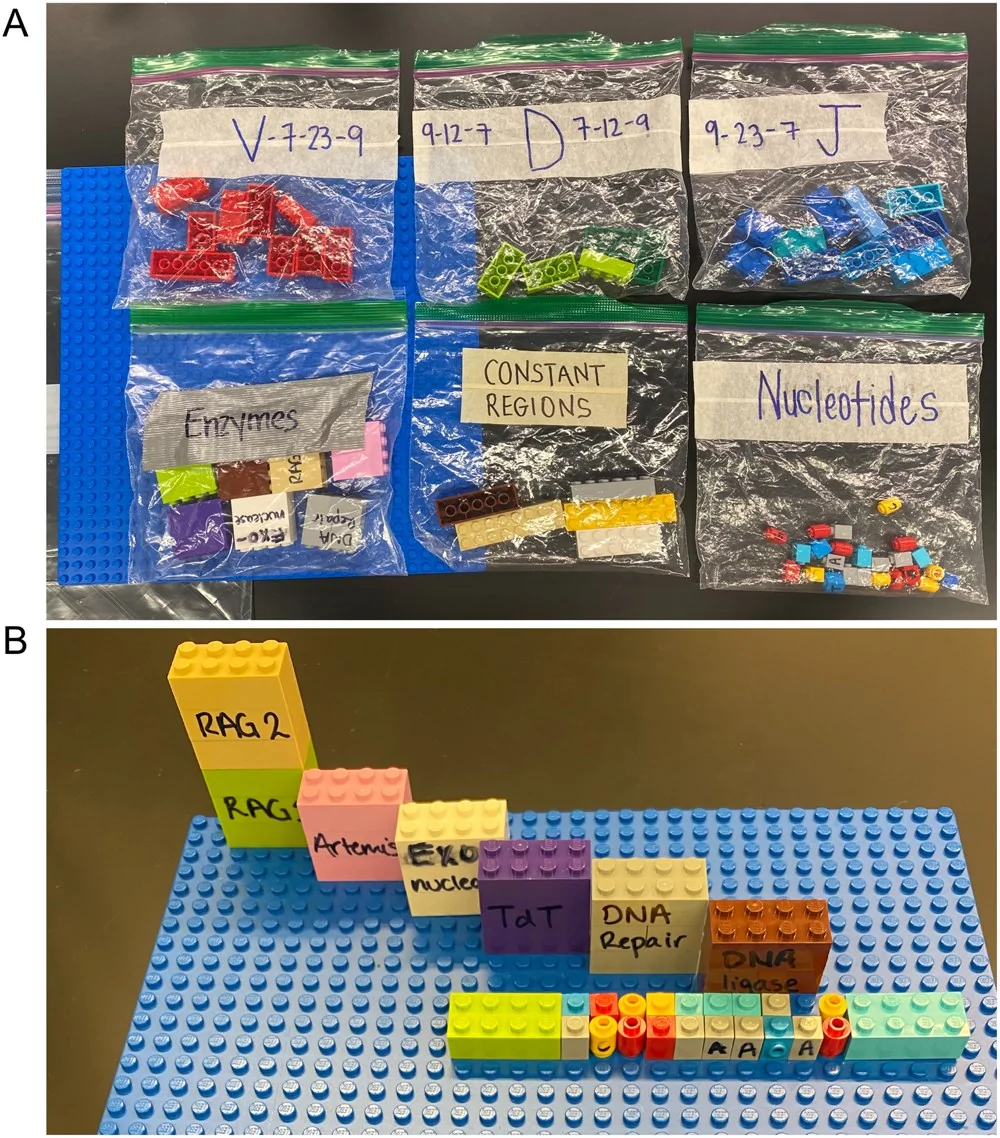
Representative image of V(D)J modeling kit and outcome of D-J recombination. Lymphocyte receptor rearrangement depicted using a modeling kit containing LEGO bricks (A), and a representative result of D-J recombination of the B-cell receptor heavy chain (B).

The modeling kits included a LEGO baseplate; 3 labeled resealable bags containing bricks representing the V, D, and J gene segments; 1 bag containing bricks representing constant region exons; 1 bag with LEGO studs representing free nucleotides; and 1 bag containing stacked LEGO pieces labeled as enzymes involved in V(D)J recombination (Fig. 1A). Detailed information about labeling, quantities, color coding, and type of bricks recommended for each component is provided in Supplemental Materials  1 and 2. Material cost for the LEGO modeling kits ranged from US$17 to $23 per kit, although costs may vary. To improve accessibility, kits can also be assembled using donated LEGO bricks or shared across classes.

In performing the activity, students used the LEGO components to simulate each step of V(D)J recombination occurring in the B-cell receptor heavy chain, following verbal instructions or written prompts (see examples in Supplemental Materials  1 and 2). In the observing condition, instructors reviewed how recombination signal sequences guide Rag1/2 enzyme binding via the 12/23 rule. Students randomly selected bricks from the D and J gene-segment bags, mimicking enzymatic nicking by Rag 1/2 and hairpin formation. Artemis was used to introduce overhangs, followed by nucleotide addition and subtraction by DNA repair enzymes, exonuclease, and TdT. Emphasis was placed on the differences between P- and N-nucleotides, and the contribution of these nucleotides to junctional diversity. This process was repeated for combining the V gene segments to recombined DJ gene segments, visually building a completely rearranged sequence and excising intervening DNA.

### Assessments

To assess student learning, all participants responded to the same open-ended essay instruction: “Describe or diagram the process of V(D)J recombination in B cells. Where applicable, include the labels for gene segments, as well as the names of major enzymes and details on what they recognize and how they change the DNA. Finally, indicate the steps that increase antigen receptor diversity.” This assignment was designed to evaluate students’ ability to recall key enzymes, explain the sequential order of molecular events, and evaluate how systems (eg genetic mechanisms, Ag receptors) connect. A common analytical scoring rubric, including a performance rating scale and numeric values, was used across all 3 institutions to ensure consistency in evaluating student responses (Table S1). For research analysis, UAB and CMU essay responses were scored by study author H.A.B. and Cal Poly responses were scored by study author M.C.G.-W. This scoring was done after the term had ended. During the term, the essay question was also graded as part of midterm assessment by the instructor and contributed to the students’ course grades. Based on defined criteria, answers were deemed excellent (100%), good (75%), fair (50%), or earning no credit (0%).

In addition to content knowledge, students completed post-activity surveys assessing their confidence in understanding V(D)J recombination and interest in the topic and activity. Surveys included a select-all-that-apply question asking about preferred learning strategies, as well as Likert-scale questions adapted from previous work on modeling in immunology education.^15^ The timing of assessments varied slightly across institutions but consistently followed the sequence of video lecture, exam, and post-activity survey.

### Statistical analysis

All statistical analyses were performed using GraphPad Prism (version 10). Group comparisons were analyzed using 1-way ANOVA and post hoc analysis, Student’s *t* tests, and χ^2^ tests, as appropriate.

## Results

### LEGO modeling activity increased learning gains

To assess student understanding of V(D)J recombination, we used a common, open-ended essay question that asked students to construct an explanation of the molecular mechanisms underlying Ag receptor diversity in B cells. This type of assessment reflects broader goals in biology education, which prioritize the application of knowledge to explain complex mechanisms rather than the recall of isolated facts. Consistent with a proposed framework to assess learning in higher education, the prompt assessed students’ ability to develop and use models, a dimension of scientific reasoning that emphasizes representing biological processes and explaining how system components interact.^3^

**Figure 2:**
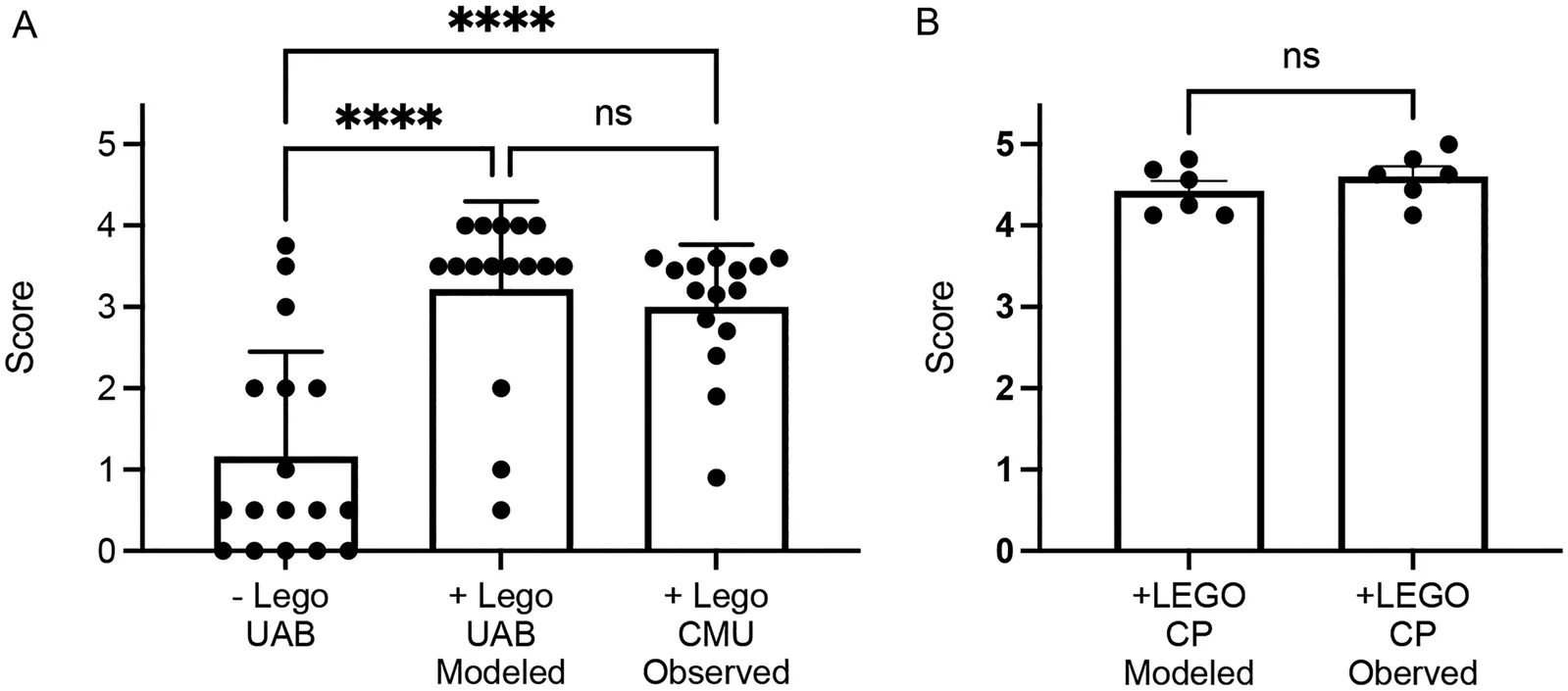
The V(D)J LEGO activity increased learning gains. Prior to the activity, all students viewed the same instructional video, and students were divided into groups as indicated. A well-defined analytical rubric was used to evaluate essay answers and scores were scaled out of 5. Symbols represent individual participants. Statistical analysis was performed via an ANOVA test and Tukey’s post-test (A) or a Student’s *t* test (B). ****P < 0.0001. Distributions pooled by educational intervention are provided in Fig. S1. UAB, University of Alabama; CMU, Central Michigan University; CP, Cal Poly.

At UAB, students self-selected into either a modeling or no-modeling group. Those who participated in the LEGO activity scored significantly higher on the essay question compared with those who opted out (Fig. 2A). A similar trend was observed when comparing results of CMU students who observed the activity during lecture with results of UAB students who elected not to participate in the LEGO activity. Using a threshold of 50% (or ≥2.5 out of 5) for a “fair” performance, we found that the proportion of students achieving or exceeding a threshold for fair performance was also higher among those who engaged with the LEGO activity. Only 3 of the 17 students (18%) in the no-LEGO group at UAB scored at or above 2.5, compared with 27 of 31 students (87%) in the UAB and CMU LEGO groups.

To confirm whether visualization of the model was sufficient for better exam performance and to directly compare a cohort exposed to the same course structure and institutional settings, we compared results of Cal Poly students who performed the activity in small groups with results of those who observed the activity. Both groups performed similarly well on the essay question, and 100% of students scored at or above 2.5 (Fig. 2B). Collectively, these findings suggest visualization of V(D)J recombination using LEGO bricks can support students in developing and using models to explain biological mechanisms.

### Students prefer learning through visuals and media

**Figure 3:**
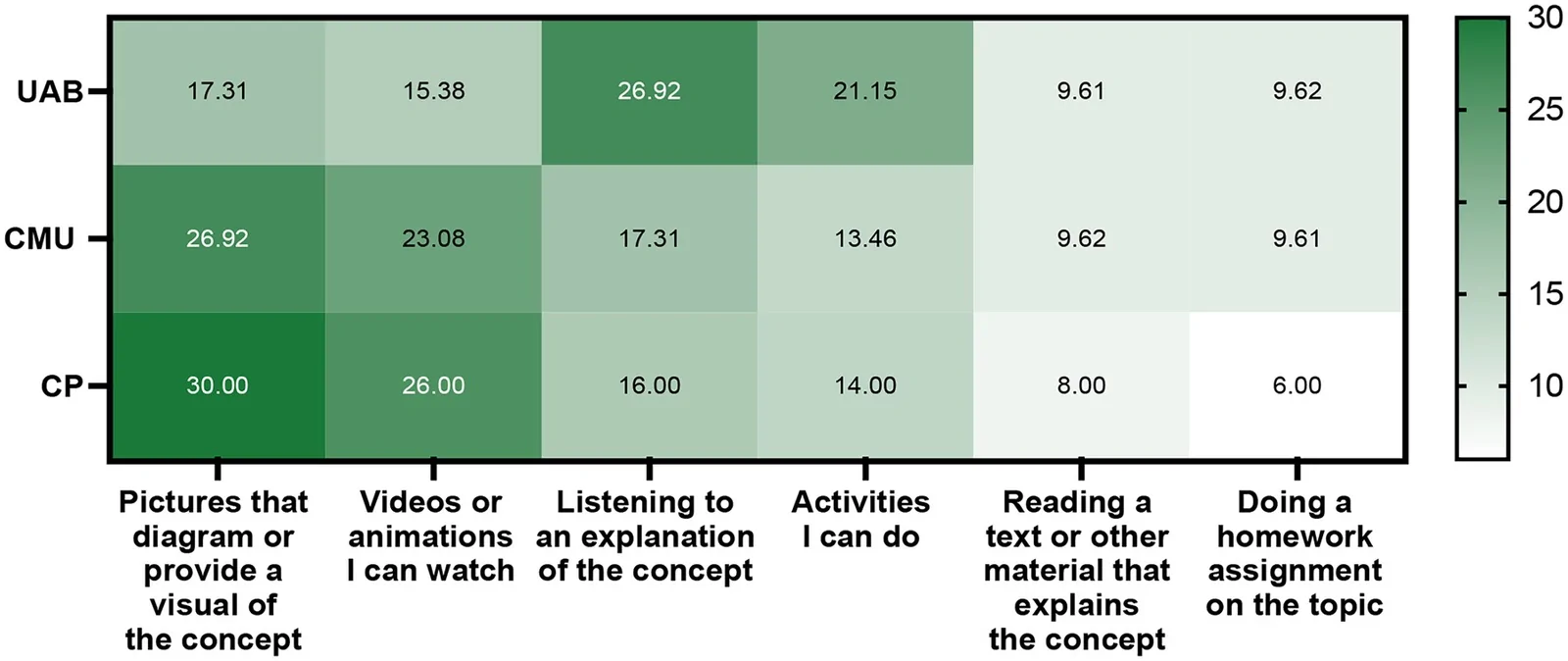
Summary of student learning preferences. Students who participated in the LEGO activity at UAB, CMU, and Cal Poly (CP) were presented this list of learning strategies and could select any they found helpful. The percentage of students who selected an activity is shown in the center of each box, and a darker green color represents a higher percentage of students.

To understand how the LEGO modeling activity aligned with students’ preferred learning modalities, we asked students to select which instructional strategies they found most helpful for understanding scientific concepts. Although preferences varied slightly by institution, visual representations, videos and animations, and verbal explanations were more frequently endorsed than were reading or homework assignments (Fig. 3). Despite the effectiveness of the LEGO activity, doing an activity did not rank among preferred learning strategies at CMU and Cal Poly. These findings indicate students prefer visual and spatial approaches to learning, but they may not initially identify active learning approaches as valuable instructional practices for understanding immunology.

**Figure 4:**
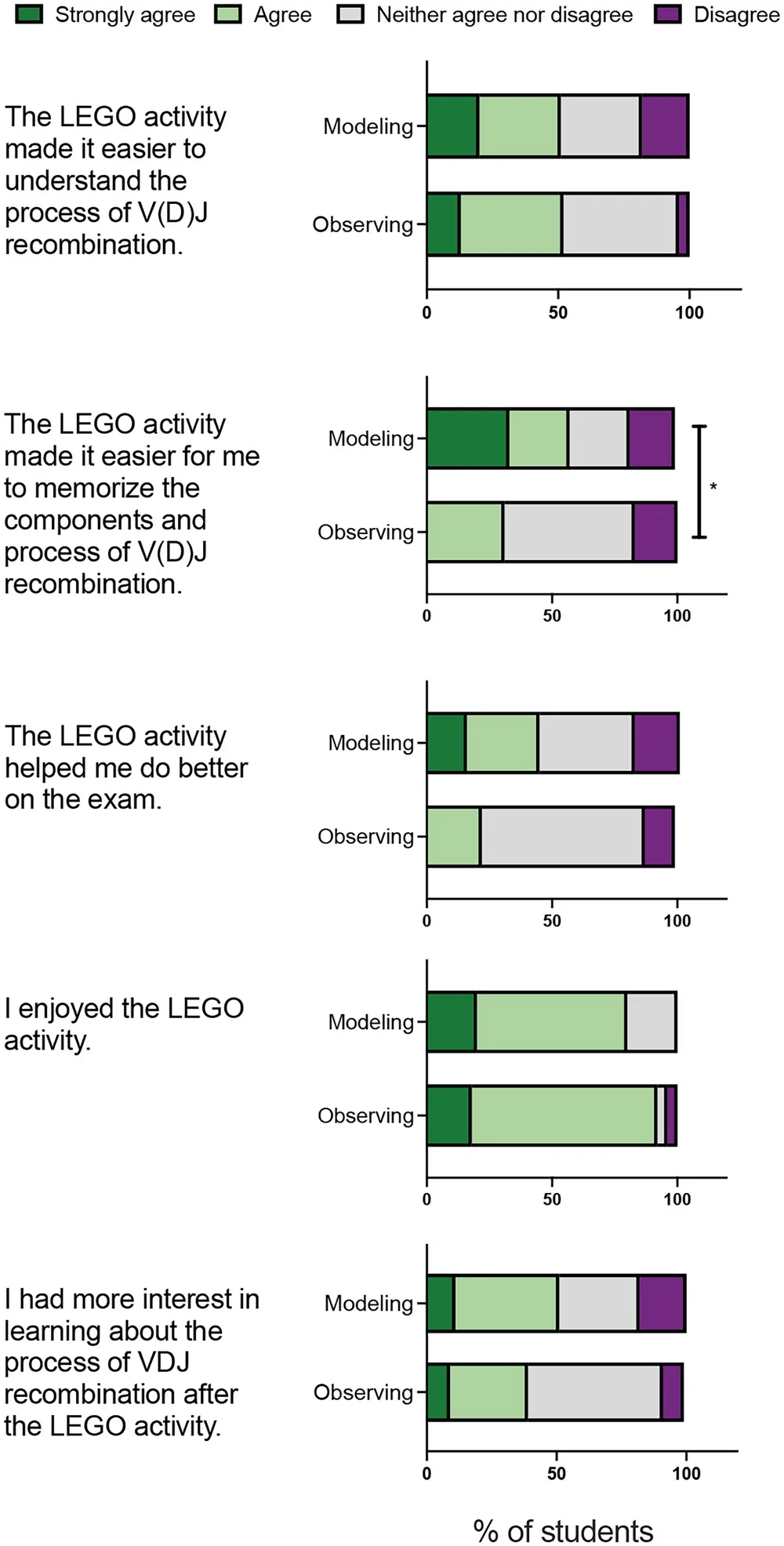
Student perceptions of the LEGO modeling activity. Students were asked to rate their agreement with 5 statements about the activity’s impact on their understanding, memorization, exam performance, and interest in V(D)J recombination compared with lecture alone. Responses were collected using a Likert scale and are color coded: green represents affirmative responses, gray represents neutral responses, and purple represents negative responses. Data are grouped by activity format. The modeling group includes students who physically performed the LEGO activity (UAB and Cal Poly), whereas the observation group includes students who watched the activity demonstration (CMU and Cal Poly). We used χ^2^ testing to compare response distributions between groups. *P < 0.05. Distributions by institution are provided in Fig. S2.

Next, we asked students about their perceptions of the LEGO modeling exercise. Half of the students agreed that the LEGO activity helped them understand the process of V(D)J recombination regardless of the activity format (Fig. 4). Responses related to memorization and exam performance revealed more variation. Students in the modeling group were significantly more likely to strongly agree that the activity helped them memorize the steps of V(D)J recombination, whereas the observation group tended to respond neutrally (*P* = 0.013). Perceived impact on exam performance tended to be more positive among the students in the modeling group, but differences between groups were not significant, and they aligned with the actual essay scores, which were similar across conditions.

Notably, students in both groups reported high enjoyment and nearly half of each class reported greater interest in V(D)J recombination after completing the LEGO exercise. Overall, these results indicate that although students may prefer to watch rather than do, hands-on modeling may enhance self-perceived retention. At the same time, students who observed the activity still demonstrated equivalent learning gains and reported high levels of enjoyment, suggesting that the cost of observation is low.

### Students feel capable of explaining a complex molecular process

**Figure 5:**
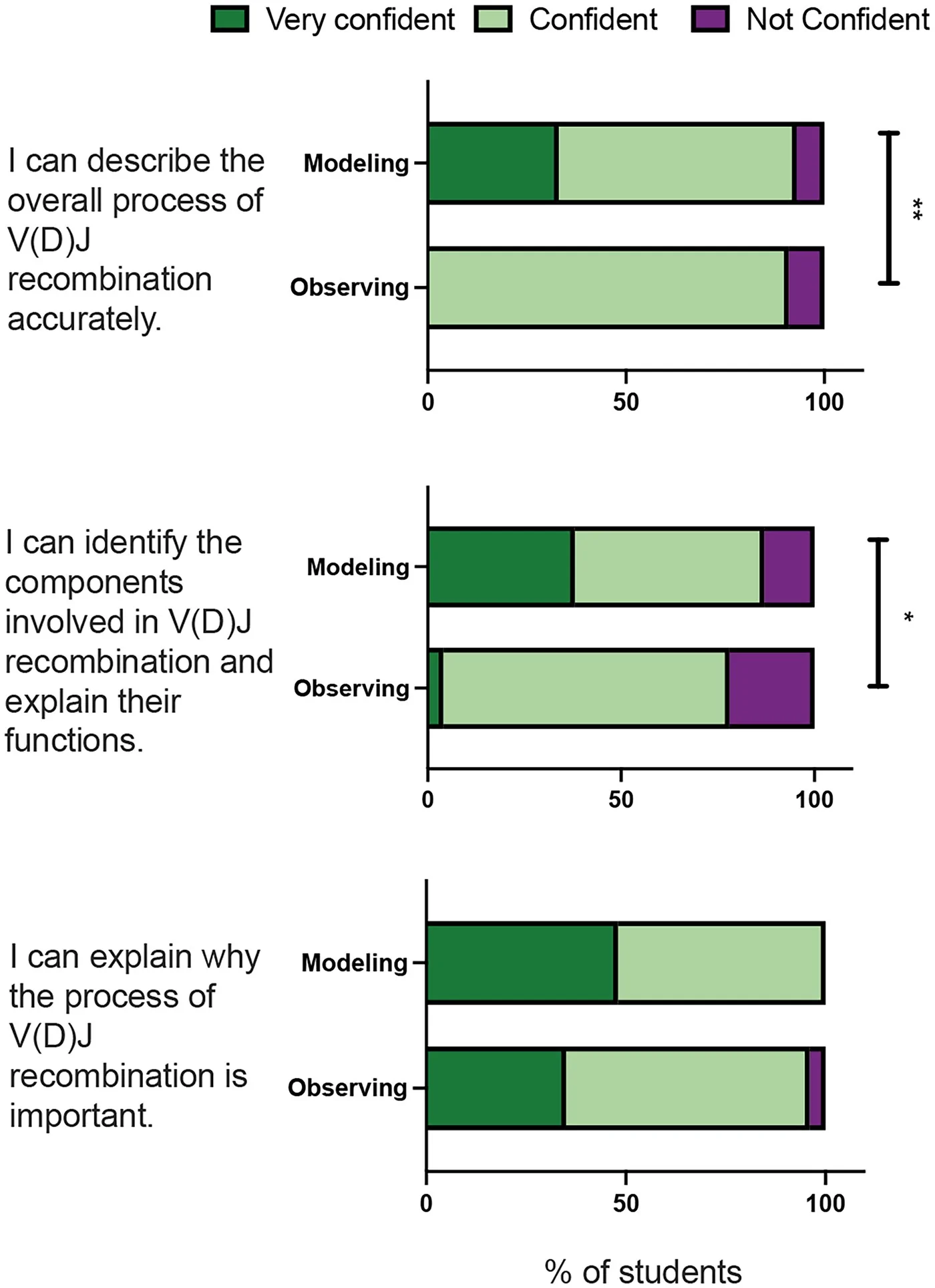
Student confidence in explaining V(D)J recombination. Students who participated in the LEGO activity were asked to rate their confidence in understanding V(D)J recombination. Responses were collected using a Likert scale and are color coded; green represents affirmative responses and purple represents negative responses. Data are grouped by activity format. The modeling group includes students who physically performed the LEGO activity (UAB and Cal Poly), and the observation group includes students who watched the activity demonstration (CMU and Cal Poly). We used χ^2^ analyses to compare response distributions between groups. *P < 0.05, **P < 0.01. Distributions by institution are provided in Fig. S3.

Lastly, we asked students about their confidence in explaining key aspects of V(D)J recombination. Across all institutions, most students reported feeling confident in their ability to sufficiently describe the overall process of V(D)J recombination accurately, identify the components involved in V(D)J recombination and explain their functions, and explain why the process of V(D)J recombination is important (Fig. 5). Students in the modeling group were significantly more likely to report a high level of confidence in their ability to describe the overall process and identify the components involved in V(D)J recombination. Overall, students were most confident in their ability to describe why the process of V(D)J recombination was important, with slightly fewer students expressing confidence in identifying molecular components and functions. Together, these data suggest the activity not only supported student comprehension but also their self-efficacy, an important factor for sustaining motivation in a conceptually dense immunology course. Because this survey was not administered to students who opted out of the activity, comparisons were limited to those who participated.

## Discussion

We show that a LEGO-based modeling activity can be an effective instructional tool for improving student understanding of V(D)J recombination. This immunological process is widely regarded as conceptually difficult, due to its molecular complexity, use of specialized nomenclature, and departure from the concept that all DNA is the same in somatic cells.^10^ Consistent with prior research demonstrating the benefits of active learning and physical modeling on learning,^1^^,^^7^^,^^15^ the LEGO activity significantly improved student performance on an exam essay question (Fig. 2). Our data across 3 institutions suggested that learning gains were comparable between students who observed the activity and those who engaged in hands-on modeling. As such, observation-based demonstrations of the LEGO model in classrooms offer a simple and cost-effective way to implement model-based instruction into immunology classrooms. This approach may be particularly useful in settings with limited resources, because materials can be donated and kits can be shared. Students’ perception of the LEGO activity and V(D)J recombination was positive overall, but students who performed hands-on modeling were more likely to strongly agree that the activity helped them memorize key aspects of the process and increased their level of confidence in describing molecular components (Figs. 4 and 5). Because perceptions of difficulty can influence motivation and persistence in STEM,^14^ incorporating hands-on modeling may enhance students’ feeling of self-efficacy and support long-term engagement with challenging molecular content.

Although the results of this study are promising, several limitations should be acknowledged. First, the courses at CMU and Cal Poly were both taught by the same instructor, which may have influenced consistency in delivery and student perception. However, this overlap also served as an internal control for the Cal Poly comparison, where only the format of the LEGO activity (modeling vs observation) differed between groups.

Second, as with many educational intervention studies, our study design introduces potential selection and response biases, both of which can influence outcomes.^16^ These biases are challenging to address retrospectively because doing so requires collection of participant characteristics, including a priori knowledge, motivation, and overall academic performance. Selection bias was potentially introduced because we did not assign students to groups. Instead, students at UAB were allowed to opt out of the activity, and it is possible that these students may have been less engaged with the course overall. Pre- and post-test assessments, as well as analysis of overall grade distributions, were not included in these IRB-approved protocols but would provide insight into the degree that this bias influenced outcomes.

Finally, although survey responses indicated a high level of enjoyment and confidence, it is important to note that the survey was voluntary and self-reported perception is not always a reliable indicator of actual learning.^17^ In our study, for example, students who observed the activity performed as well as those who modeled it, despite reporting lower agreement with the statement that the LEGO activity helped them to memorize parts of the immunological process. However, because students enjoyed the activity and because 87% of students who did the activity scored at or above a fair (ie 50%) level of understanding, we recommend incorporating this activity in immunology classrooms.

The feelings associated with modeling, such as increased enjoyment, interest, and confidence, may be especially valuable in courses in which cognitive overload and student disengagement are common. Interest and motivation are key drivers of persistence in STEM,^14^ and students are more likely to retain concepts and grow professionally when they are personally invested in the learning process.^18^ LEGO bricks were chosen for this exercise due to their ease of accessibility; however, LEGO is an extremely popular toy brand, and its familiarity, along with play-based association, may be related to positive perception of the activity. Interestingly, students who observed the activity were less likely to strongly agree that the activity improved memorization or exam performance. Further work could clarify what dimensions of perception-, retention-, or learning-specific objectives are most shaped by tactile engagement; however, in this instance, observation alone supported learning, confidence, and interest.

Ultimately, this study contributes to the growing body of literature supporting the use of physical models to teach conceptually challenging content in biology. We showed that the activity improved student performance and confidence and that it can be flexibly implemented in immunology classrooms. Thus, this exercise would be a useful addition to an immunology teaching toolkit.

## Supplementary Material

vlag043_Supplementary_Data

## Data Availability

The datasets for this study are available upon request.
