# Supplementary material for: Increasing learning, confidence, and retention in the immunology classroom with LEGO
Source: Immunohorizons. 2026 Aug 13;10(8):vlag043. doi: 10.1093/immhor/vlag043 (PMC13471997; doi:10.1093/immhor/vlag043)
Supplement: Supplementary file 1 [file vlag043_supplementary_data.pdf]

Supplemental Materials 1. LEGO modeling instructor guide

A. Materials

1. One baseplate
2. Three zip-lock bags with bricks representing V, D, and J gene segments
   - Label the V bag as “V 7-23-9” and fill with ~10 4x2 red bricks
   - Label the D bag as “9-12-7 D 7-12-9” and fill with ~5 4x2 green bricks
   - Label the J bag as “9-12-7 J” and fill with ~10 4x2 blue blocks
3. One zip-lock bag with LEGO studs representing “free nucleotides”
   - T (shown in blue)
   - A (shown in gray)
   - C (shown in yellow)
   - G (shown in red)
4. One zip-lock bag with bricks representing enzymes. Duplos or three stacked 4x2 bricks works well for enzymes. On the bricks, write in permanent marker:
   - RAG1/2, Artemis, Exonuclease, DNA repair enzymes, DNA ligase, and Tdt
5. Optional
   - One zip lock with bricks representing constant regions – 5 6x2 bricks for each isotype
   - Addition enzymes (e.g., AID)
   - 1x1 studs for uracil

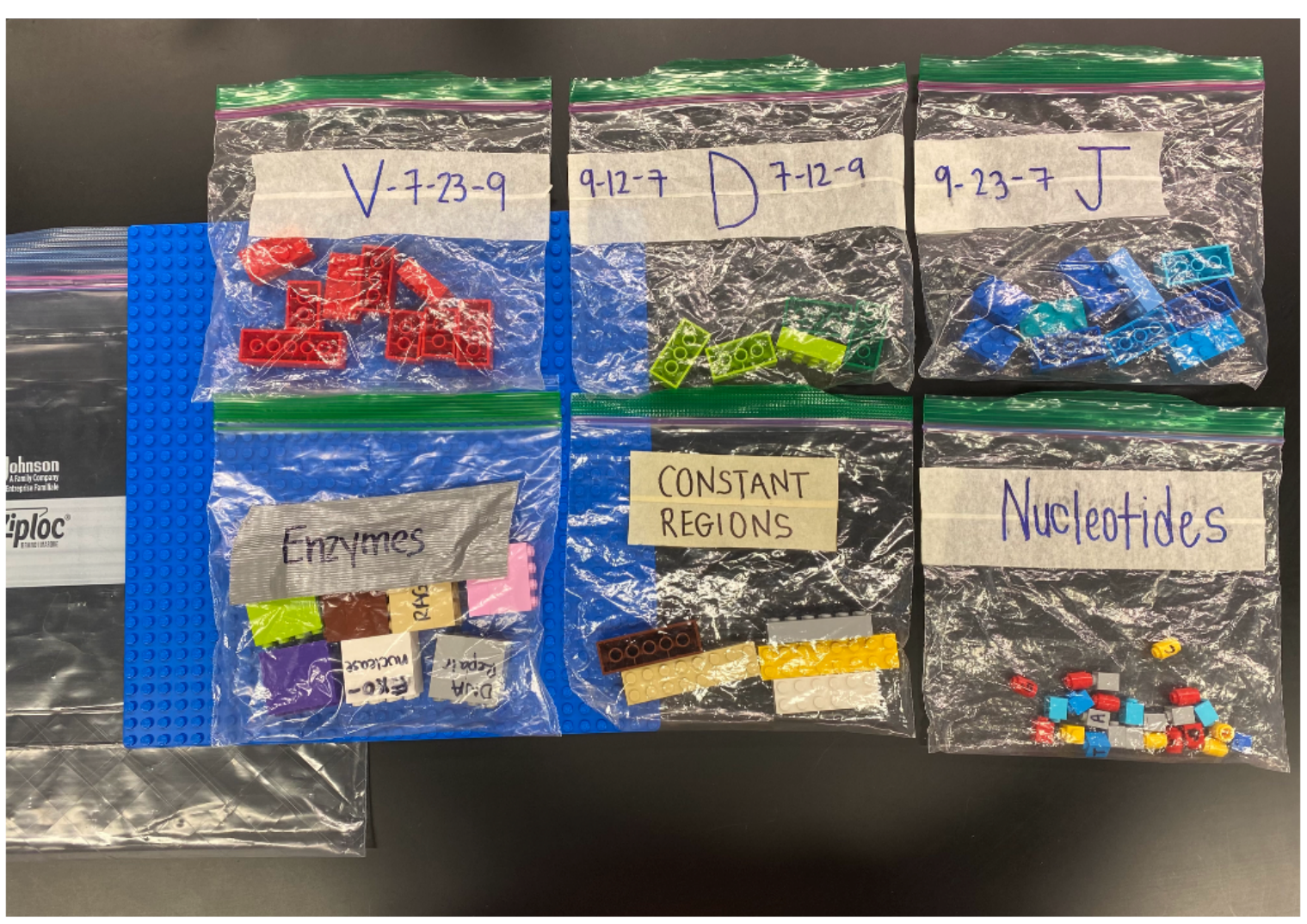

Instructor Guide Image 1. Image of kit contents

B. Class outline

1. Prior to the activity, introduce the material through instructional video, reading, or lecture
2. Recommended: To assess preparedness, assign students 3-5 question quiz with recall questions about V(D)J recombination (**5 minutes**)
3. Refresh material with mini-lecture (**10 minutes**)
4. Model V(D)J rearrangement with LEGOs
    - For the hands-on version of the activity, provide students with printed instructions and the LEGO kits (**40 minutes**)
    - For the observed model, see description below (**20 minutes**)
5. Recommended: wrap up with multiple-choice formative assessment via polling software or A-D cards. (**10 minutes**)

C. V(D)J Modeling Activity

1. Set up the document camera or other similar technology (e.g., Airplay) to display and describe the kit components
2. Explain what the V, D, and J bags represent
    - Emphasize that **each brick in the bag represents an individual gene segment** in your genome, and that only one of the gene segments will eventually encode for a piece of the BCR heavy chain.
    - Describe the labels on the bag, which represent the **recombination signal sequences (RSS)** flanking each gene segment. In other words, a brick represents the gene segment with its flanking RSS sequence. The RSS sequences contain a conserved nonamer, a 12 or 23 base-pair spacer sequence, and a highly conserved heptamer sequence.
3. Ask students to name the enzyme that recognizes the RSS flanking gene segments.
    - This should be **Rag1/2** enzyme brick.
    - Emphasize the importance of the RSS display on the bags (**IMAGE 2A**):
        - the **nonamer sequence** helps recruit the enzyme (specifically Rag1) to the correct DNA location
        - the **spacer** (either 12 or 23) has two critical functions
            1. to position the heptamer sequence precisely within the RAG complex for accurate DNA cleavage
            2. to enforce the **12/23 rule**, which ensures that gene segments recombine is the proper order (e.g., D to J, or V to D) and that the D segments are not skipped.
        - the **heptamer sequence** is highly conserved and it is where the Rag1/2 enzyme cuts to initiate recombination

4. Ask a student to reach into the D and J bags and grab a random brick. Emphasize that joining these gene sequences happens 1st, follows the 12/23 rule, and is **random**. Randomly combining V(D)J gene segments contributes to combinatory **diversity** of the BCR.
5. Place each brick on the baseplate about 10 spaces apart (**IMAGE 2B**). Explain that RAG1/2 performs a **single stranded nick at the end of the heptamer sequence**, which generates a 3' hydroxyl group. The 3' hydroxyl attacks a 5' phosphate on the opposite strand, creating a DNA **hairpin** in the coding region. Show the hairpin structure with 4 studs as demonstrated in **IMAGE 2C**.
6. Recap that the RAG1/2 enzymes located the RSS, introduces a nick at the heptamer, and formed DNA hairpins in the coding regions. You may want to note that this step also liberates the intervening DNA, including the RSS, which are joined in the signal joint. (This part is not depicted in the LEGO model)

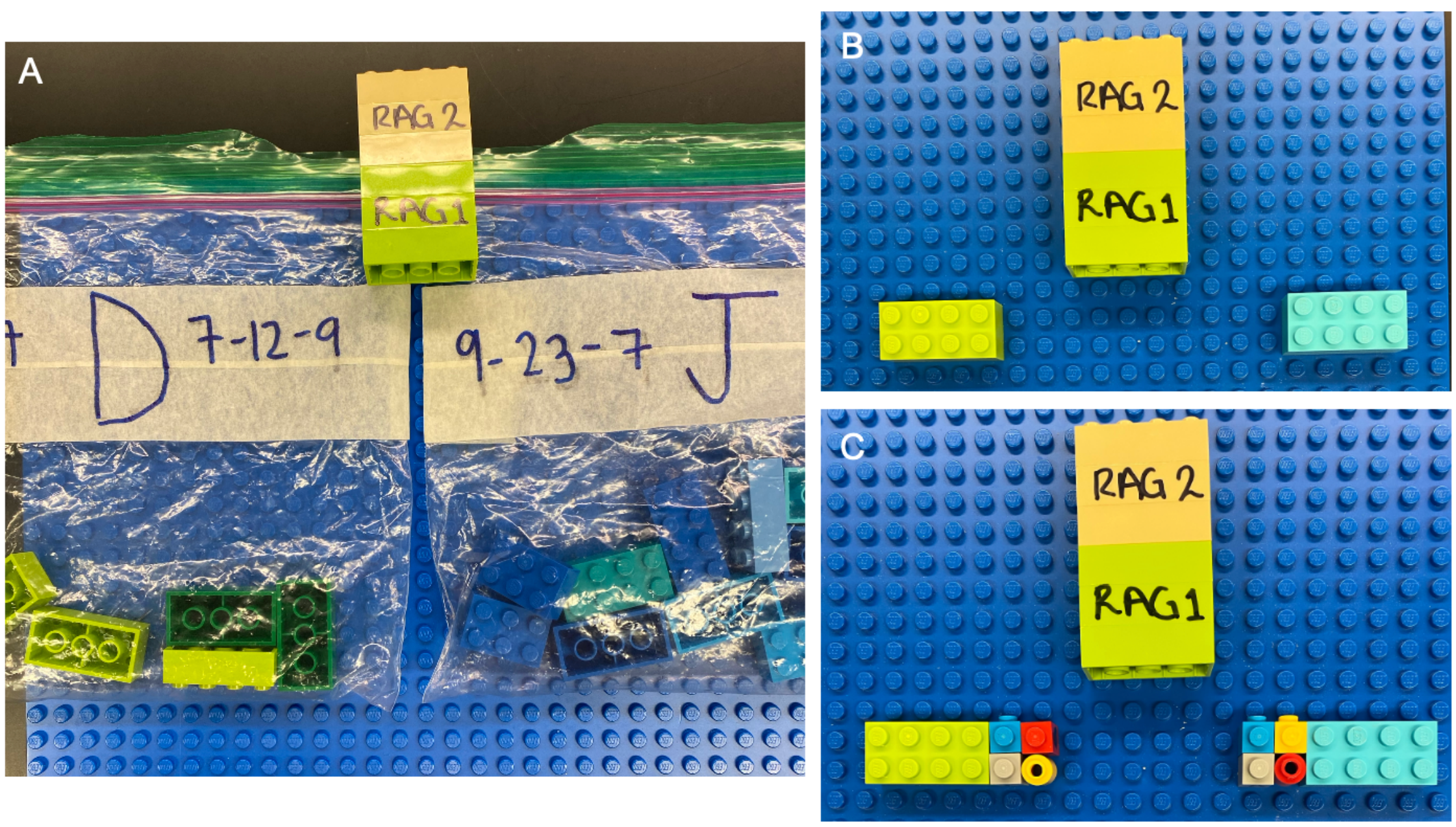

Instructor Guide Image 2. Recombination until hairpin formation.

7. Ask students to name the enzyme that opens the hairpin.
    - The correct answer is the **Artemis** enzyme brick.
    - Explain that Artemis opens the hairpin at the coding end. Artemis can cleave at various points in the hairpin, and this results in variation at the DNA ends…
        - If Artemis cuts at the midpoint, it generates blunt ends.
        - More commonly, Artemis cuts off-center, producing overhangs (single-stranded DNA) at the coding ends
    - Model for the students what it would look like if Artemis **cuts off-center to create an overhang** (**IMAGE 3A, B and C**). Explain that this overhang can be filled in or trimmed (at random) contributing to more diversity in the BCR.
8. Next, explain that when Artemis cuts the hairpin asymmetrically, it creates single-stranded **overhangs**.
    - If **DNA repair enzymes** immediately fill-in the complementary strand **without further trimming or modification**, the result is a palindromic sequence. The are called **palindromic (P)-nucleotides**. Show this process in the LEGO model (**IMAGE 3D**).
    - You may also want to note that the light chain is modified only by P-nucleotides (as just demonstrated), but this is not depicted in the LEGO model.

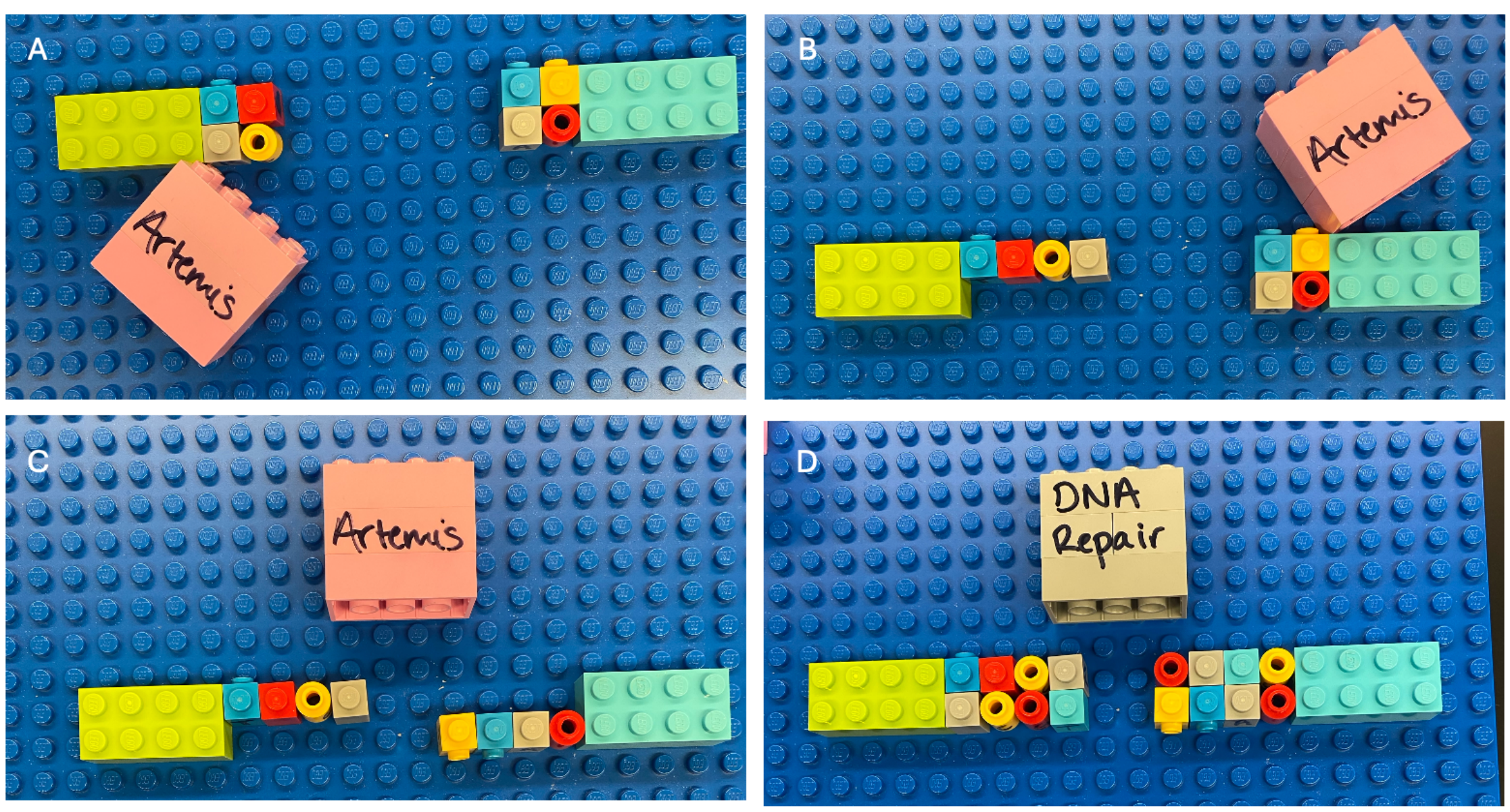

Instructor Guide Image 3. Artemis action and addition of P-nucleotides

9. Reset the overhang to show that in the heavy chain, the overhang is modified further before addition of P-nucleotides (**IMAGE 4A**).
10. Explain that in the heavy chain, the overhang is further modified by **exonuclease and TdT**. Locate these enzymes and set them on the model.
    - Explain that exonuclease removes nucleotides (at random) from the overhang. Model this process by removing 1-3 studs from the overhang (**IMAGE 4B, C, and D**).
    - Explain that Tdt is an enzyme expressed during recombination of the heavy chain. It random nucleotides to the overhang and these nucleotides are called N-nucleotides for their 'non-templated' nature. Model this process by adding 2-4 studs to the overhang (**IMAGE 4E and F**).
    - The random modification of the overhangs at the coding ends helps generate enormous diversity of the BCR.

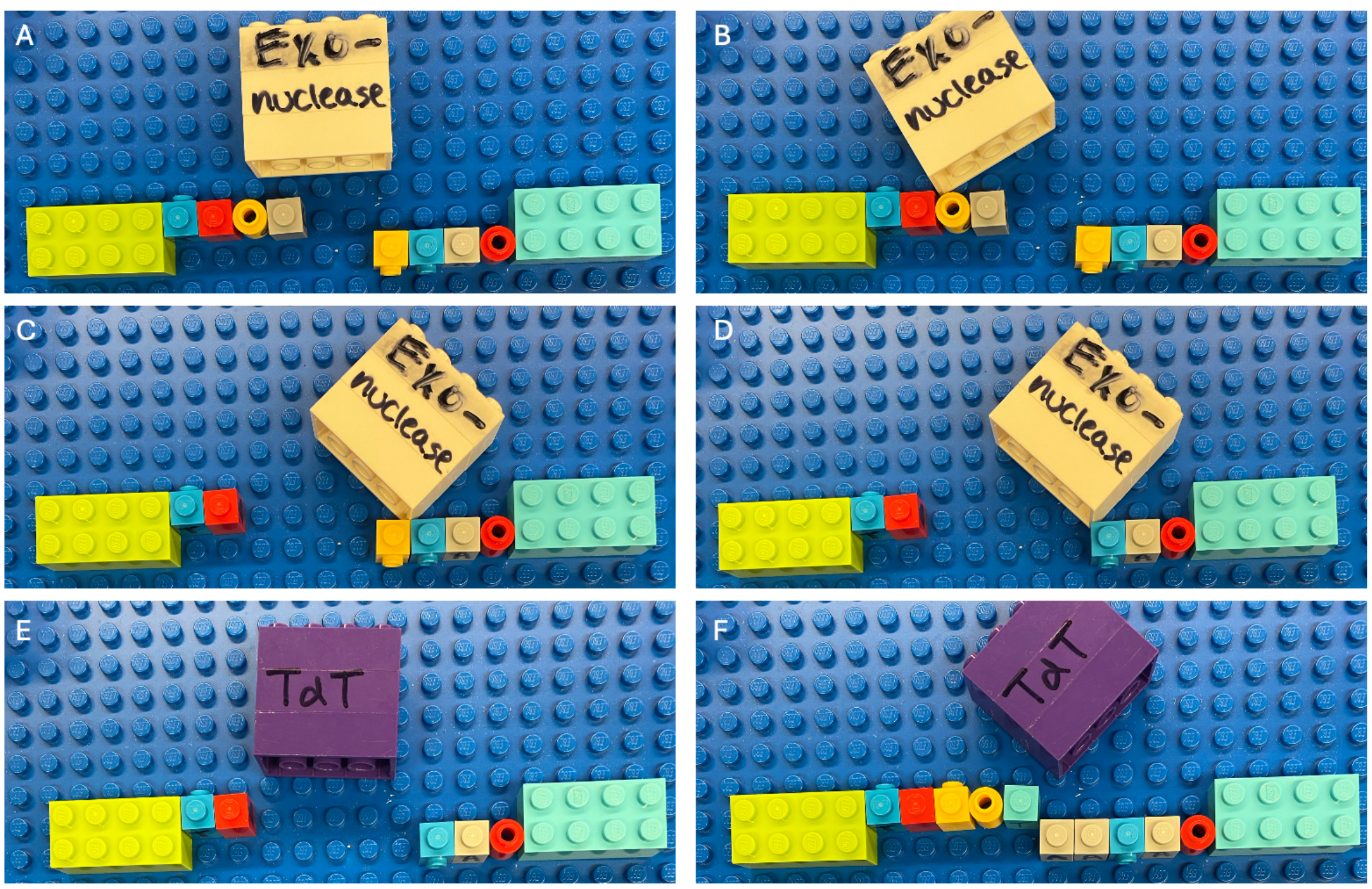

Instructor Guide Image 4. Editing by Exonuclease and Tdt

- Instructional note: students often inquire about whether an overhang is necessary for exonuclease and TdT activity, and whether they must always act sequentially as illustrated in the model. In reality, neither exonuclease nor TdT strictly requires an overhang. Both enzymes can act on blunt or staggered ends, but the overhang was selected to re-

enact the textbook diagram. Additionally, while the LEGO model shows a simplified sequential process – first exonuclease trimming, then TdT addition – in the cell, these steps may occur in varied order or even at the same time, and not all ends undergo both modifications. The model emphasizes key concepts, but actual recombination events are stochastic and contribute to diversity through imprecise and flexible processing.

11. After modeling exonuclease and TdT, you are ready to fill in the gaps. Find the **DNA repair enzymes** and **DNA ligase bricks** and lay them under the document camera. Explain that the coding joints from the D and J gene segments are connected by this DNA repair machinery.
    - First, show that **DNA repair enzymes** (or DNA polymerases) fill in any single-stranded gaps between the two ends (**IMAGE 5A and B**).
    - Then, model the action of **DNA ligase**, which seals the final phosphodiester bond to create a continuous DNA strand (**IMAGE 5C**).
    - Recap that gene segments, asymmetrical cleavage by Artemis, exonuclease activity, and TdT all contributed to random diversity in the coding region.
    - Emphasize the result in a joined DJ segment. The process is repeated again, joining V to DJ, and completing the coding joint (**IMAGE 6A and B**).
    - Instructional note: A limitation of the LEGO model is the fixed distance between bricks on the baseplate, which may unintentionally suggest that the spacing between gene segments is fixed or uniform in the genome. In reality, the physical distance between gene segments can very significantly within the chromatin. The RAG1/2 complex brings together two distant RSS through looping and synapsis, and the actual positioning is dynamic and flexible, not linear or evenly spaced. You may want to explain briefly that this model simplifies a 3D process into a linear 2D format for clarity.
12. Optional: explain that the coding joint (VDJ) forms one exon of the BCR heavy chain gene (**IMAGE 6**). This exon encodes for the **variable region of the heavy chain**. This gene is now ready to be transcribed into **pre-mRNA**, that includes sequences for:
    - The joined VDJ exon
    - Remaining downstream J gene segments
    - exons encoding the constant regions, including Cμ and C∂.

During alternative splicing, the cell selects the VDJ exon and either the Cμ or C∂ exon to include in the mature mRNA. This determines whether the BCR will be of the IgM or IgD isotype.

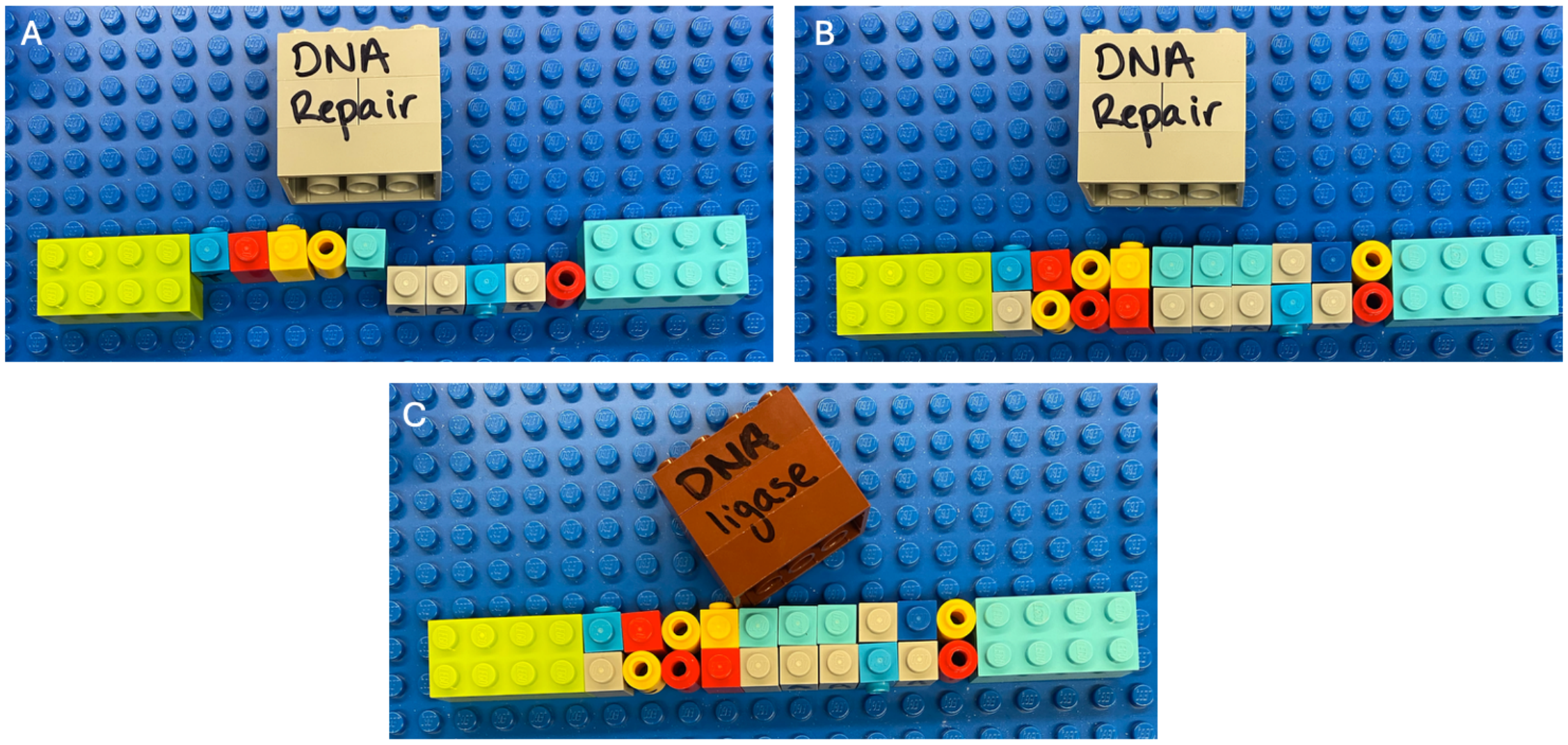

Instructor Guide Image 5. Sealing the D and J coding joints

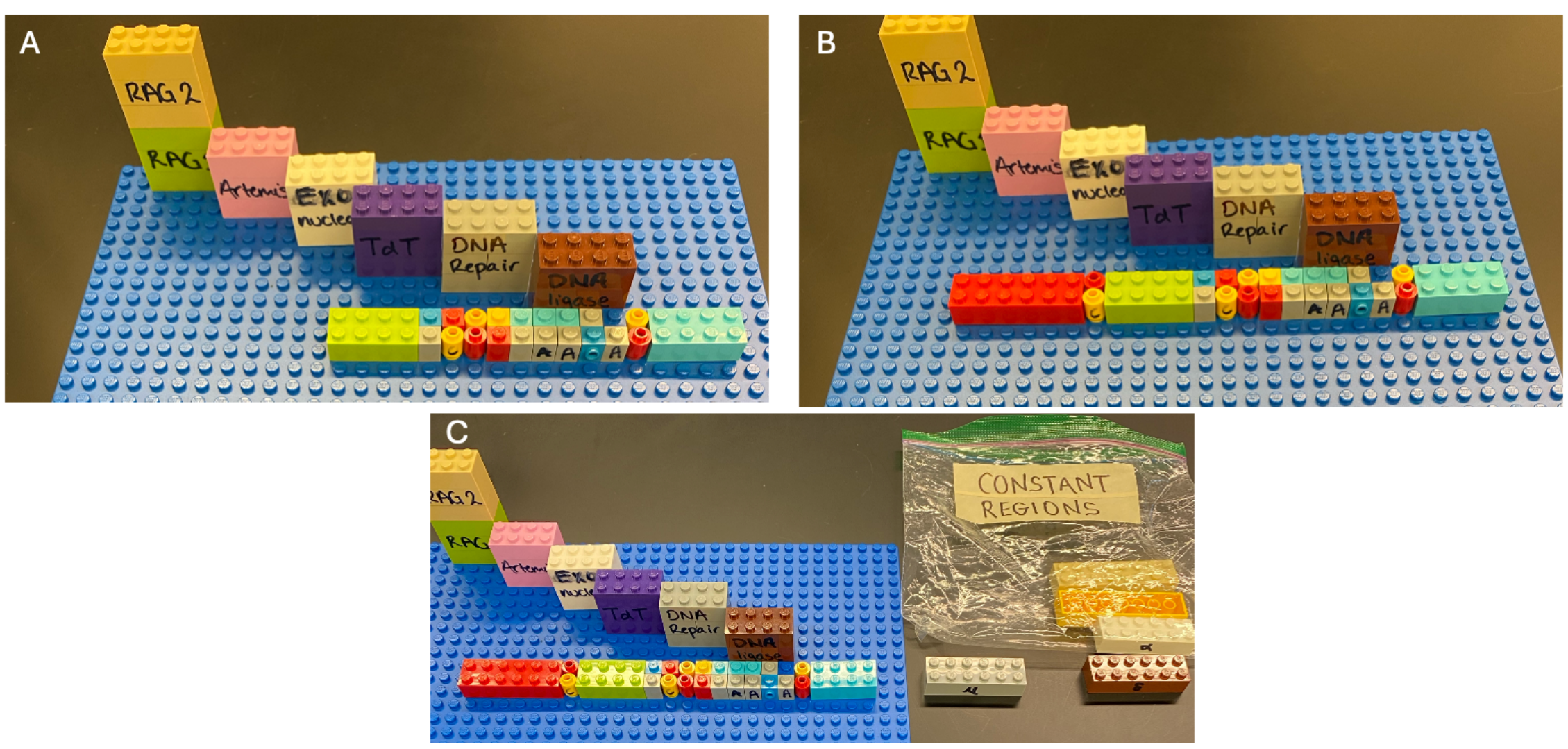

Instructor Guide Image 6. LEGO representation of Heavy chain Exons

Supplemental Table 1. V(D)J Exam Question Rubric and Question.

Criteria	Excellent (100%)	Good (75%)	Fair (50%)	No credit (0%)
**Gene segments** **(1 | 0.75 | 0.5 | 0)**<br>1. Accurately labels V, D, and J gene segments and constant region.<br>2. Indicates that DJ (or 2+3) joins first	All correct	Missing one	Missing two	Missing more than 2
**Rag Action** **(1.5 | 1.125 | 0.75 | 0)**<br>1. Specifies that Rag1/2 binds to RSS as a first step.<br>2. Includes detail about the 12/23 base pair spacers *or* heptamer/nonamer (RSS) sequences that guide RSS binding.<br>3. Specifies that Rag nicks one strand of dsDNA	All details	Correct placement & calls out enzyme, but missing 1 detail	Correct placement & calls out enzyme, but missing 2 details	Missing 2+ or more details or omits the step
**Hairpin formation** **(1 | 0.75 | 0.5 | 0)**<br>1. Places hairpin formation after Rag action.<br>2. Explains mechanism for hairpin formation (e.g., the free hydroxyl attacks phosphate on opposite strand)	All details	Correct placement and calls out hairpin, but missing mechanism	Calls out hairpin, but is misplaced and missing mechanism	Omits the step
**Artemis Action (1 | 0.75 | 0.5 | 0)**<br>1. Places Artemis/overhang formation after hairpin<br>2. Explains that Artemis opens the hairpin and produces different overhangs	All details	Correct placement. Missing <u>either</u> overhangs or Artemis	Misplaced and missing <u>either</u> overhangs or Artemis	Missing 2+ or more details or omits the step
**P-nucleotide addition** **(1.5 | 1.125 | 0.75 | 0)**<br>1. Places P-nucleotide addition after overhang generation.<br>2. Explains that DNA repair enzymes add P-nucleotides<br>3. Specifies that this step introduces new sequences- or - adds diversity	All details	Correct placement. Missing one of: enzyme, p-nucleotides, addition of diversity	Missing two of the following: placement, enzyme, p-nucleotides, addition of diversity	Missing 3 or more details or omits the step
**Exonuclease**** **(1.5 | 1.125 | 0.75 | 0)**<br>1. Places exonuclease after P-nucleotide addition.<br>2. Explains that exonucleases trims off some the overhang nucleotides randomly<br>3. Specifies that this step changes existing sequences- or - adds diversity	All details	Correct placement. Missing one of: enzyme, mechanism, addition of diversity	Missing two of the following: placement, enzyme, mechanism, addition of diversity	Missing 3 or more details or omits the step
**Tdt and N-nucleotides** **(1.5 | 1.125 | 0.75 | 0)**<br>1. Places Tdt after exonuclease action<br>2. Explains that Tdt adds non-templated (N) nucleotides to the overhangs<br>3. Specifies that this step changes existing sequences- or - adds diversity	All details	Correct placement. Missing one of: enzyme, n-nucleotides, addition of diversity	Missing two of the following: placement, enzyme, n-nucleotides, addition of diversity	Missing 3 or more details or omits the step
**Ligase action** **(1 | 0.75 | 0.5 | 0)**<br>1. Places this step last<br>2. Explains that DNA ligase now joins modified D and J segments together	All details	Correct placement. Missing name of enzyme or what occurs.	Missing two of the following: placement, name of enzyme, what occurs.	Missing 3 or more details or omits the step

*I usually let them use one extra asterisk without taking off points.
**Some text books show this step occurring after TdT, so this may need to be revised on a class-by-class basis.
**Sometimes students tell me about the light chain and/or excision of the signal joint, but I do not grade or check for accuracy.
*** I allow for the misspelling of enzymes, as long as they are ‘close enough’

Starting with the gene segments below, diagram or describe the process of V(D)J recombination in B cells. Where applicable include the following:

(a) labels for the gene segments and other regions and which join first;
(b) names of major enzymes and details on what they recognize <u>and</u> how they change the DNA
(c) indicate the steps that increase antigen receptor diversity (can place an asterisk by these steps). *

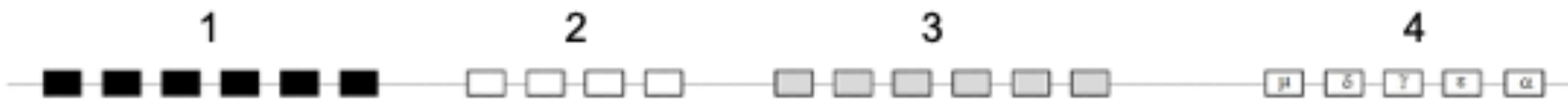

Supplemental Figure 1

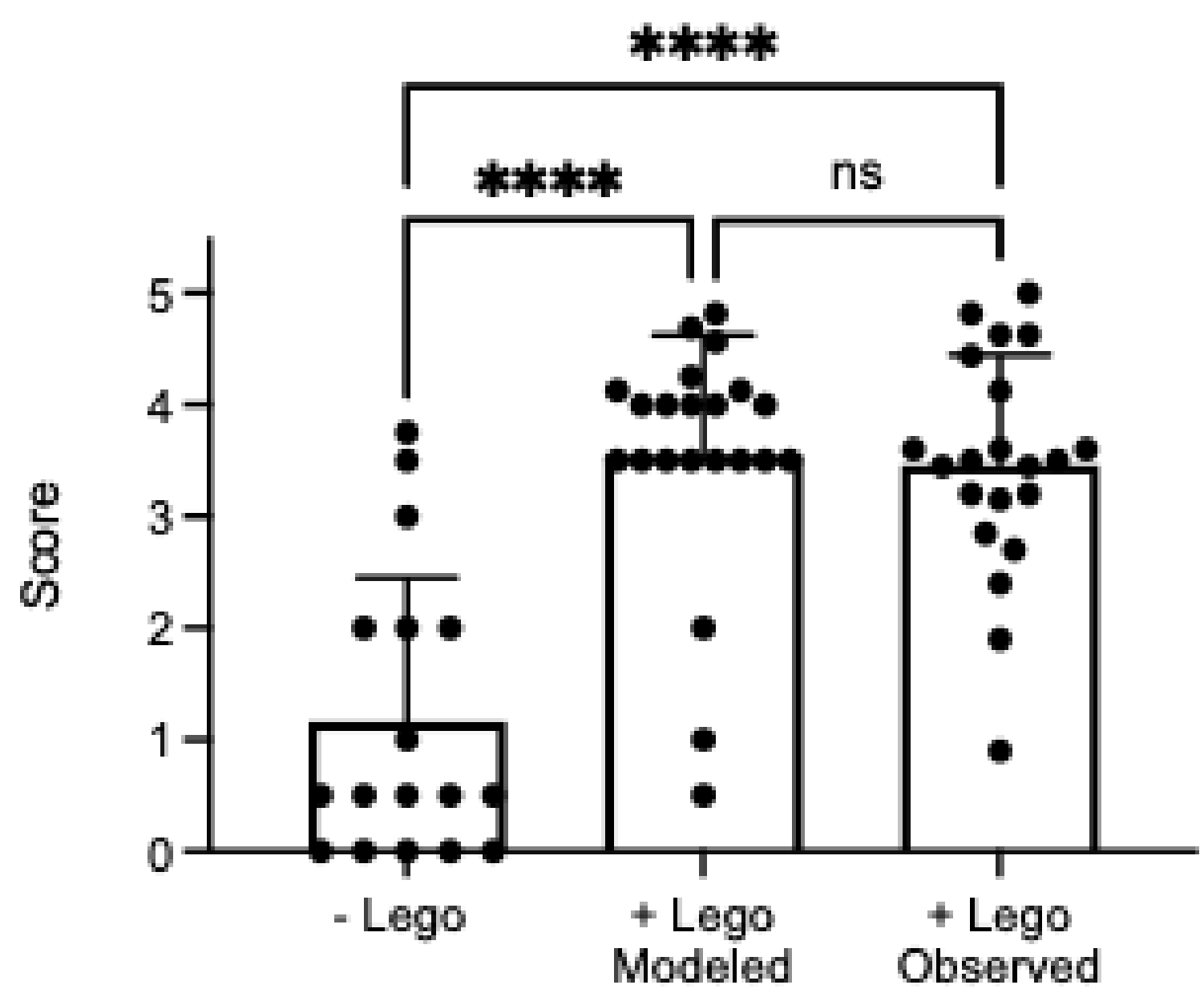

Supplemental Figure 1. Pooled analysis of student performance following the LEGO based activity. Prior to the activity, all students viewed the same instructional video. Students were grouped according to intervention format: no activity (UAB students who opted out of the LEGO activity), modeling (UAB and Cal Poly students who performed the activity), or observation (CMU and Cal Poly students who observed the activity).. A well-defined analytical rubric was used to evaluate essay answers and scores were scaled out of 5. Symbols represent individual participants. Statistical analysis was performed via an ANOVA test and Tukey's post-test (A, ****$p<0.0001$)

Supplemental Figure 2

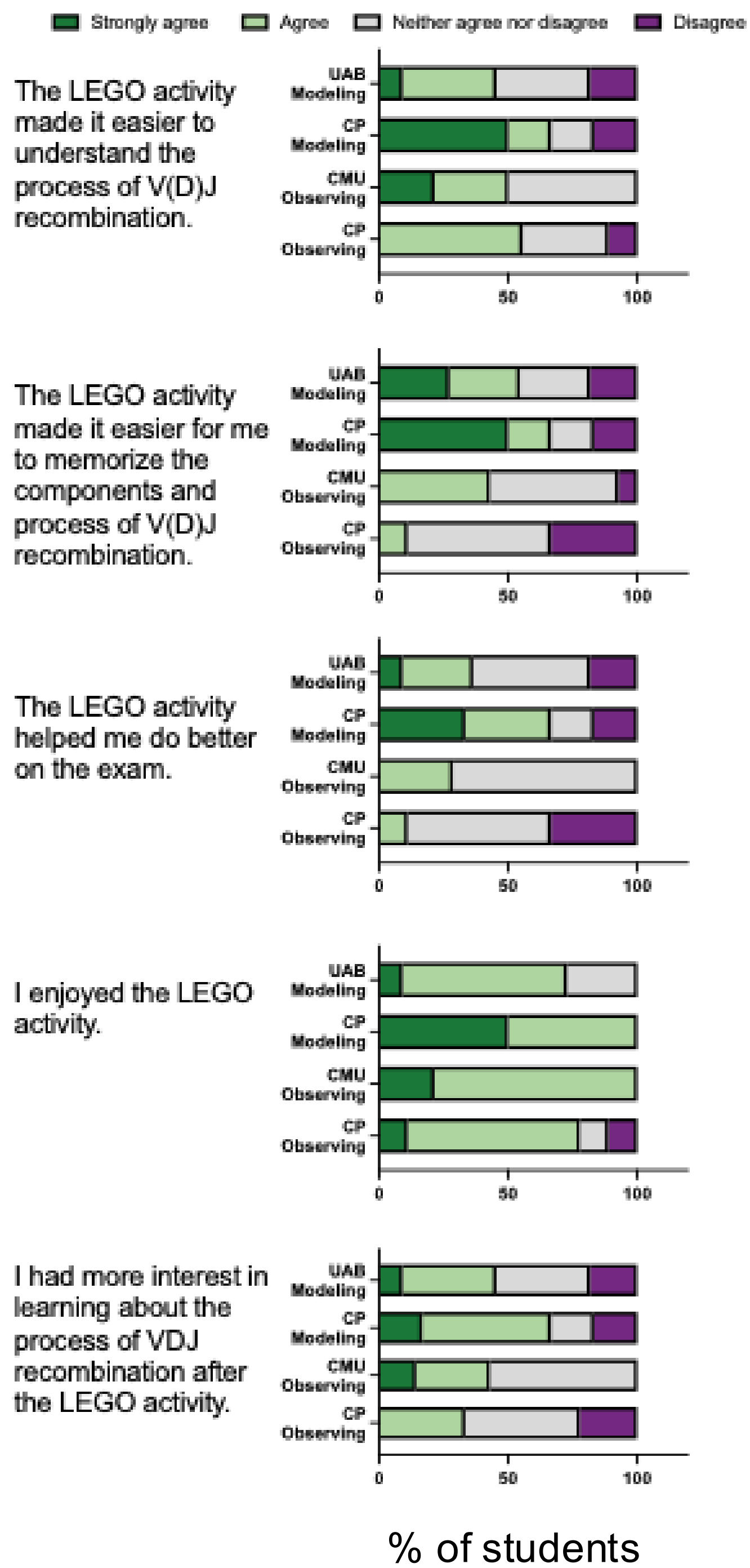

Supplemental Figure 2. Student perceptions of the LEGO modeling activity by institution. Responses to the survey statements described in **Figure 4** are shown separately for UAB, CMU, and Cal Poly. Responses are grouped according to activity format (doing the activity vs. watching).

Supplemental Figure 3

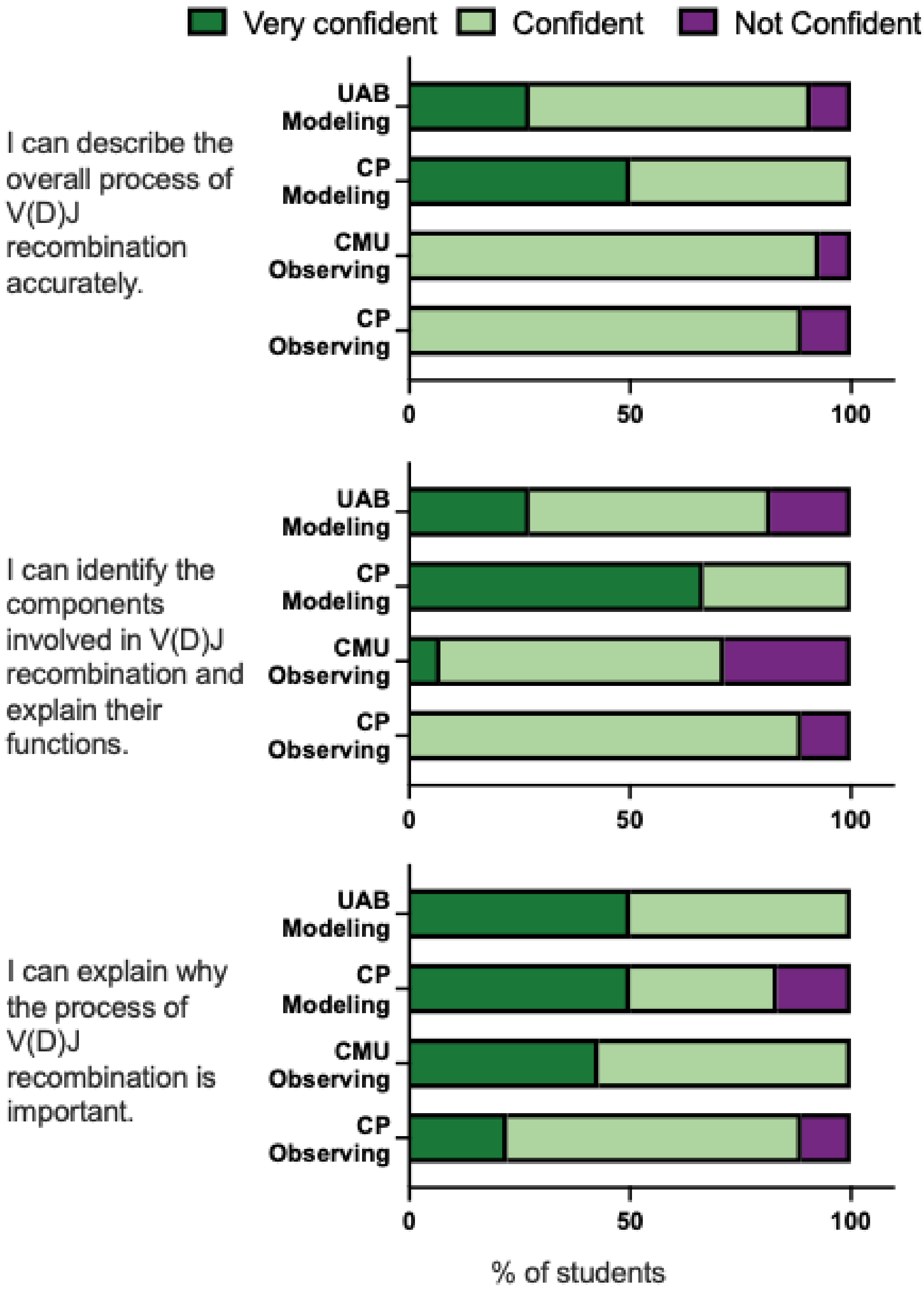

Supplemental Figure 3. Student confidence in explaining V(D)J recombination by institution. Responses to the survey statements described in **Figure 5** are shown separately for UAB, CMU, and Cal Poly. Responses are grouped according to activity format (doing the activity vs. watching).
